# Reaction-based energy level modulation of a cyclometalated iridium complex for electrochemiluminescent detection of formaldehyde†

**DOI:** 10.1039/d3ra06936b

**Published:** 2023-11-01

**Authors:** Hyun Seung No, Jong-In Hong

**Affiliations:** a Department of Chemistry, Seoul National University 1 Gwanak-ro, Gwanak-gu Seoul 08826 Korea jihong@snu.ac.kr

## Abstract

Formaldehyde is a toxic compound present in both the environment and living systems, and its detection is important due to its association with various pathological process. In this study, we report a new electrochemiluminescence (ECL) probe based on a cyclometalated iridium complex (IrHAA) for the selective detection of formaldehyde. The homoallylamine moiety in IrHAA reacts with formaldehyde, undergoing a 2-aza-Cope-rearrangement reaction to form a formyl group. Significant changes in the electronic properties and molecular orbital energies of the iridium complex through the functional group transformation result in enhanced ECL and radiometric phosphorescence changes, enabling the quantitative and selective detection of formaldehyde. The energetic requirements for ECL sensing were investigated, highlighting the importance of the excited state energy for achieving efficient ECL. The sensing mechanism was elucidated using NMR spectroscopy and MALDI-TOF analysis.

## Abstract 2

An ECL formaldehyde probe was developed by modulating the LUMO energy level of the probe before and after the reaction with formaldehyde.

## Graphical abstract

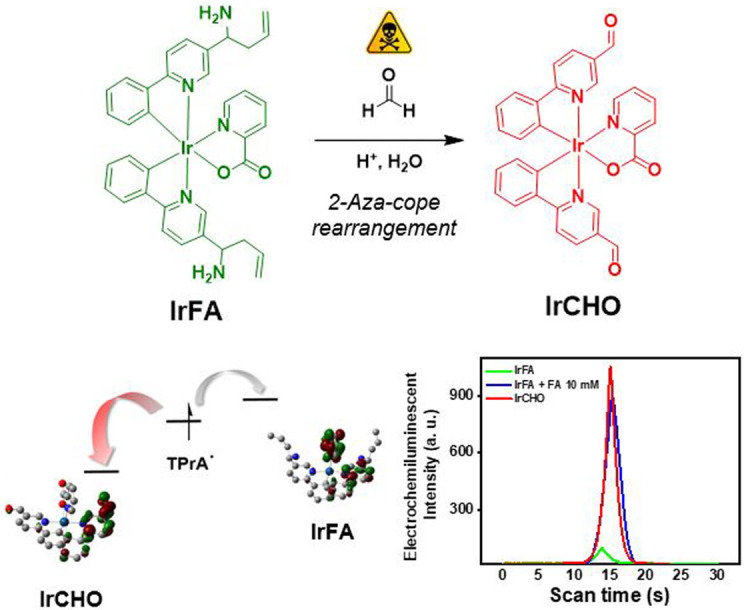

## Introduction

Formaldehyde is the simplest form of aldehyde present in both biological and environmental systems.^1^ It finds wide-ranging applications in laboratories and industries, such as protein fixation, preservation agents, and as an ingredient in resin and other materials. Formaldehyde is a highly toxic chemical that has been classified as a Group 1 human carcinogen by the International Agency for Research on Cancer (IARC) in 2004. Exposure to formaldehyde can cause various symptoms, including irritation, respiratory disorders, and headaches. Moreover, prolonged exposure to formaldehyde has been associated with the development of fatal diseases such as cancer and Alzheimer's disease.^2,3^ Formaldehyde is also an environmental pollutant that poses a significant threat to the aquatic ecosystem and wildlife. For these reasons, it is crucial to develop a real-time method for quantifying and monitoring the level of formaldehyde in the environment for the safety and well-being of individuals. Indeed, a variety of formaldehyde detection methods have been reported in the literature, including colorimetric,^4^ high-performance liquid chromatographic,^5^ radiometric,^6^ and mass spectrometric analysis.^7^ However, all these analytical methods have limitations such as bulky equipment, high cost, and lack of applicability for real-time quantitative determination.

Electrochemiluminescence (ECL) is a light emission process resulting from excited state molecules generated through electron transfer reactions between radical species formed near an electrode.^8,9^ ECL does not require a bulky external light source and therefore provides not only low background signal and high sensitivity, but also can be used to build inexpensive and miniaturized instruments. Especially, “co-reactant ECL” which uses a co-reactant as a redox mediator, enables highly sensitive detection of a target analyte using a low concentration of the probe molecule. Co-reactant ECL can be employed for the development of real-time monitoring devices for diagnostic molecules and environmentally toxic compounds.^10^ ECL luminophores require excellent electrochemical stability as well as desirable photophysical properties due to the inherent instability of the radical species formed during electron transfer. Tris(bipyridine)ruthenium(ii) complex (Ru(bpy_3_)^2+^) has been widely utilized as an ECL luminophore due to its high ECL efficiency and ability to meet the aforementioned requirements.^8–10^ Nevertheless, the low energy level of the metal-centered state in ruthenium complexes induces substantial non-radiative decay from the metal-to-ligand charge transfer (MLCT) emission. This low energy level also presents challenges in altering the luminescence wavelength through ligand modification.^11^ In contrast, cyclometalated iridium complexes are known to exhibit excellent luminescence efficiency. Moreover, the luminescence wavelength and redox potential of these complexes can be adjusted by choosing the appropriate ligand.^12^ It has been reported that a chelating ligand in iridium complexes has a significant impact on their photophysical properties, electronic properties, and ECL efficiency of the complex.^13,14^ We have previously reported several iridium complexes that exhibit higher ECL efficiencies compared to Ru(bpy_3_)^2+^. These iridium complexes have been successfully combined with molecular probes for ECL detection of various target molecules.^15–17^

In this study, we developed a cyclometalated iridium complex-based probe (IrHAA) that can selectively detect formaldehyde using ECL as well as phosphorescence. The reaction of formaldehyde with the homoallylic amine moiety of IrHAA produced *N*-methylene homoallylic iminium which subsequently underwent the cationic 2-aza-Cope rearrangement reaction followed by hydrolysis to generate a formylated iridium complex, IrCHO.^18,19^ The electron withdrawing formyl group of the resulting IrCHO stabilizes its LUMO (lowest unoccupied molecular orbital) energy level, causing ECL enhancement and enabling the quantitative detection of formaldehyde.

## Experimental

### Materials and instruments

All the chemical reagents used in this study were purchased from Sigma-Aldrich (Sigma-Aldrich Corp., MO, USA), Tokyo Chemical Industry (TCI, Tokyo, Japan), or Alfa (Alfa Aesar, MA, USA) and were used without additional purification. The organic solvents were purchased from Samchun Chemical (Co., Seoul, Korea). Flash column chromatography was performed using silica gel 60 (230–400 mesh) from SILICYCLE (Quebec, Canada). Deuterated solvents required for nuclear magnetic resonance (NMR) spectra were purchased from Cambridge Isotopic Laboratories (Cambridge, MA, USA). All the ^1^H and ^13^C NMR spectra were recorded using a 400 MHz DD2MR400 Agilent NMR system or a Varian 500 MHz NMR system. High-resolution mass spectra (HRMS) were obtained at the National Center for Inter-University Research Facilities (NCIRF) using a JMS-700, 6890 series instrument (JEOL, Japan) with fast atom bombardment (FAB) in positive mode. Matrix assisted laser desorption/ionization time-of-flight (MALDI-TOF) mass spectra were obtained using a Microflex instrument from Bruker Daltonics. JASCO V-730 spectrometer was used for measurement of ultraviolet-visible (UV-vis) absorption spectra. The photoluminescence spectra were recorded using a JASCO FP-8300 spectrometer. The electrochemical properties were examined using cyclic voltammetry or differential pulse voltammetry techniques with a CH Instruments 650 B (CH Instruments, Inc., TX, USA). All the redox potential values were referenced with respect to the ferrocene/ferrocenium (Fc/Fc^+^, 1 mM) redox couple. The ECL intensities were measured using a low-voltage photomultiplier tube (PMT) module (H-6780, Hamamatsu Photonics K.K., Tokyo, Japan) with an arithmetic unit.

### Experimental methods

Stock solutions of iridium complexes (2 mM) were prepared in dimethyl sulfoxide (DMSO) and subsequently diluted with either deionized water or acetonitrile (CH_3_CN). Formaldehyde (37%, 10 ∼ 15% methanol for a stabilizer) was purchased from Sigma-Aldrich Corp., and was diluted with deionized water or CH_3_CN (ACROSS organic, spectrometer grade) to prepare the stock solution. Each sample was prepared by the addition of 10 μL of the stock solution to a 1.99 mL solution containing 10 μL of the analyte. The final volume of each sample was adjusted to 2 mL. Trifluoroacetic acid (TFA) was purchased from Samchun Chemicals and diluted with CH_3_CN to prepare a 0.1% TFA solution in CH_3_CN. The acetate buffer (10 mM) was prepared by diluting a mixture of sodium acetate (NaOAc) and acetic acid (HOAc) with deionized water. Stock solutions of various analytes (10 mM) were prepared by dissolving in acetate buffer solution. ECL was generated using a cyclic voltammetry scan with a scan range of 0 to 1.5 V *vs.* Ag/AgCl and a scan rate of 0.1 V s^−1^. For the ECL experiments, a 250 μL flow cell was used and directly mounted on the PMT module. Tetrabutylammonium perchlorate (TBAP) from TCI (Japan) was employed as the supporting electrolyte, and tripropylamine (TPrA) from Sigma-Aldrich Corp. (MO, USA) was used as the co-reactant. The platinum (Pt) working electrode (rod and disk) was polished with 0.05 μm alumina from Buehler (IL, USA) on a felt pad before and after the measurements. The electrode was then sonicated in a 1 : 1 (v/v) mixture of distilled water and ethanol for 4 min and dried using ultrapure nitrogen gas for 1 min. All the samples for electrochemical and ECL measurements were freshly prepared immediately before conducting the experiments.

### Synthesis of iridium complexes (Scheme 1)

The synthesized iridium complexes were fully characterized using ^1^H and ^13^C NMR spectroscopy, as well as HRMS analysis (ESI, Fig. S11–S19).†

**Scheme 1:**
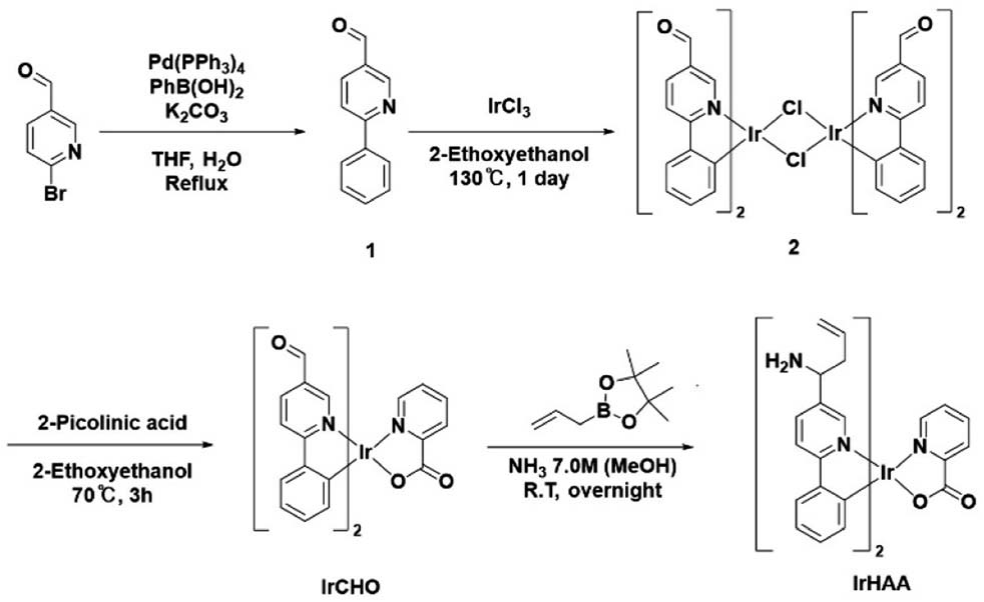
Synthetic scheme of iridium complexes.

#### Synthesis of 1 (ref. 20 and 21)

A mixture of phenylboronic acid (671 mg, 5.50 mmol), 6-bromo-3-pyridinecarboxaldehyde (930 mg, 5.00 mmol), tetrakis(triphenylphosphine)palladium (289 mg, 0.0250 mmol), and potassium carbonate (2.07 g, 15.0 mmol) in a 1 : 1 mixture of deaerated THF (50 mL) and deionized water (50 mL) was refluxed overnight under a nitrogen atmosphere. The resulting mixture was cooled to room temperature and then treated with ethyl acetate (50 mL) and water (50 mL). The organic layer was extracted with the addition of brine and dried over Na_2_SO_4_, filtered, and concentrated *in vacuo*. The residue was purified by silica gel column chromatography using a mixture of hexane and ethyl acetate (1 : 1 v/v) as the eluent to give compound 1 as an off-white solid (778 mg, 4.25 mmol, 85%). ^1^H NMR (400 MHz, CDCl_3_) *δ* 10.14 (s, 1H), 9.13 (s, 1H), 8.23 (dd, *J* = 8.3, 1.9 Hz, 1H), 8.10–8.06 (m, 2H), 7.91 (d, *J* = 8.2 Hz, 1H), 7.51 (d, *J* = 7.2 Hz, 3H).

#### Synthesis of 2 (ref. 20–22)

A mixture of IrCl_3_ (227 mg, 0.760 mmol) and compound 1 (350 mg, 1.91 mmol) in 2-ethoxyethanol (24 mL) and deionized water (8 mL) was refluxed for 24 h. After cooling to room temperature, cold water (30 mL) was poured into the reaction mixture. The suspended solution was filtered and thoroughly washed with cold methanol to yield compound 2 as a red powder (308 mg, 0.260 mmol, 68%). ^1^H NMR (400 MHz, DMSO-d_6_) *δ* 10.40 (s, 1H), 10.11 (s, 1H), 10.05 (s, 1H), 9.96 (s, 1H), 8.50–8.39 (m, 4H), 7.90 (dd, *J* = 19.6, 7.8 Hz, 2H), 6.96–6.75 (m, 4H), 6.35 (d, *J* = 7.7 Hz, 1H), 5.71 (d, *J* = 7.7 Hz, 1H).

#### Synthesis of IrCHO

A mixture of compound 2 (120 mg, 0.101 mmol), 2-picolinic acid (36.6 mg, 0.295 mmol), and Na_2_CO_3_ (53.0 mg, 0.500 mmol) in 2-ethoxyethanol (6 mL) was refluxed at 70 °C for 3 h. The hot reaction mixture was the filtered through a plug of Celite® using ethyl acetate as the solvent. The solvent was removed under reduced pressure. The residue was purified by silica gel chromatography using a mixture of dichloromethane and methanol (50 : 1, v/v) as the eluent. The product was triturated with acetone and hexane. The resulting precipitate was then filtered to give IrCHO as a red powder (81.0 mg, 0.120 mmol, 59%). ^1^H NMR (500 MHz, CD_3_CN) *δ* 9.99 (s, 1H), 9.75 (s, 1H), 9.09 (s, 1H), 8.28–8.17 (m, 5H), 8.03 (d, *J* = 13.0 Hz, 2H), 7.87 (t, *J* = 6.7 Hz, 2H), 7.78 (d, *J* = 4.9 Hz, 1H), 7.50 (d, *J* = 5.7 Hz, 1H), 7.04–6.97 (m, 2H), 6.90–6.82 (m, 2H), 6.46 (d, *J* = 7.3 Hz, 1H), 6.25 (d, *J* = 7.5 Hz, 1H). ^13^C NMR (101 MHz, CD_3_CN) *δ* 189.53, 189.31, 173.02, 172.27, 152.46, 151.64, 151.36, 151.11, 150.07, 148.89, 148.73, 143.31, 143.00, 138.75, 137.48, 137.26, 132.75, 132.69, 131.33, 130.82, 130.79, 130.57, 128.81, 128.01, 126.66, 126.27, 122.05, 121.72, 119.42, 119.19; HRMS (FAB) *m*/*z*: [M + H]^+^ observed 680.1163 (calculated for C_30_H_21_IrN_3_O_4_ [M + H]^+^ 680.1161).

#### Synthesis of IrHAA

A solution of IrCHO (30 mg, 0.044 mmol) in methanolic ammonia solution (7.0 M, 1.5 mL) was stirred at room temperature for 2 h. Then, allylboronic acid pinacol ester (58.0 μL, 0.385 mmol, 8.75 equiv.) was added to the resulting suspended solution and stirred at room temperature overnight. The solution was exposed to air for 30 min to remove excess ammonia. After that, deionized water (3 mL) was added, and the organic layer was extracted with ethyl acetate (10 mL), then dried over Na_2_SO_4_. The residue was purified by silica gel column chromatography using a mixture of dichloromethane and methanol (1 : 1) as the eluent. The purified product was recrystallized with acetone and hexane, and the resulting precipitate was filtered to yield IrHAA (10 mg, 0.013 mmol, 30%) as a yellow powder. ^1^H NMR (400 MHz, CD_3_CN) *δ* 8.56 (s, 1H), 8.19 (d, *J* = 7.0 Hz, 1H), 8.01 (d, *J* = 8.6 Hz, 2H), 7.90 (d, *J* = 8.2 Hz, 1H), 7.84 (d, *J* = 8.2 Hz, 1H), 7.73 (dd, *J* = 23.3, 4.1 Hz, 4H), 7.46 (d, *J* = 4.1 Hz, 2H), 6.91 (dd, *J* = 15.8, 7.7 Hz, 2H), 6.76 (dd, *J* = 14.7, 7.4 Hz, 2H), 6.31 (d, *J* = 7.7 Hz, 1H), 6.16 (d, *J* = 7.6 Hz, 1H), 5.73–5.57 (m, 2H), 5.00–4.89 (m, 4H), 4.05 (d, *J* = 6.3 Hz, 1H), 3.83 (d, *J* = 6.6 Hz, 1H), 2.35 (dd, *J* = 15.5, 9.2 Hz, 4H), 2.11 (d, *J* = 1.1 Hz, 4H). ^13^C NMR (101 MHz, CD_3_CN) *δ* 166.63, 166.57, 165.91, 148.48, 147.20, 146.82, 144.73, 144.38, 138.17, 136.37, 136.16, 136.13, 134.72, 134.69, 134.65, 132.48, 132.28, 129.35, 129.33, 128.90, 128.44, 128.41, 127.75, 127.69, 124.29, 123.88, 121.40, 121.07, 118.84, 118.70, 118.57, 118.52, 52.58, 52.35, 43.31, 43.23; HRMS (FAB) *m*/*z*: [M + H]^+^ observed 762.2422 (calculated for C_36_H_35_IrN_5_O_2_ [M + H]^+^ 762.2420).

## Results and discussion

### Design strategy

**Scheme 2:**
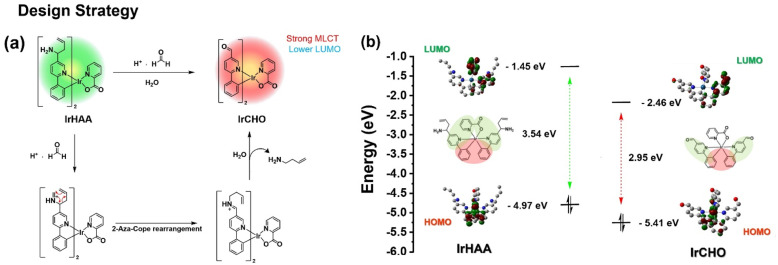
(a) Schematic representation of the design strategy of the formaldehyde probe (IrHAA) involving the cationic 2-aza-Cope rearrangement, (b) comparison of energy levels of iridium complexes obtained from DFT calculations. Method: B3LYP/GEN(6-31G(d,p) + LALN2DZ).

It is known that the LUMO electron density is distributed over the pyridyl moiety in the case of iridium complexes having a phenylpyridine as the main ligand. We expected that the LUMO energy level can be modulated before and after the reaction of IrHAA with formaldehyde, because the electron-donating homoallylic amine moiety is converted to the electron-withdrawing formyl moiety after the reaction of IrHAA with formaldehyde. The strong electron withdrawing effect of the formyl group decreases the LUMO energy level of IrCHO, resulting in a bathochromic shift in the phosphorescence emission (Scheme 2). Accordingly, the phosphorescence of IrCHO obtained after reaction of IrHAA with formaldehyde represents ratiometric change compared to IrHAA.

One important requirement for ECL generation is the thermodynamically favourable electron transfer from the TPrA radical (co-reactant) to the iridium complex (luminophore).^14,15^ The free energy difference between the singly occupied molecular orbital (SOMO) energy level of a TPrA radical and the LUMO energy level of an oxidized IrCHO plays a crucial role in efficient electron transfer for ECL generation.^23^ Based on this principle, we devised IrHAA where the LUMO energy level is lowered by the formyl group of IrCHO generated upon the reaction of IrHAA with formaldehyde, facilitating the electron transfer and turn-on response in ECL (*vide infra*).

### Photophysical properties of iridium complexes

**Fig. 1:**
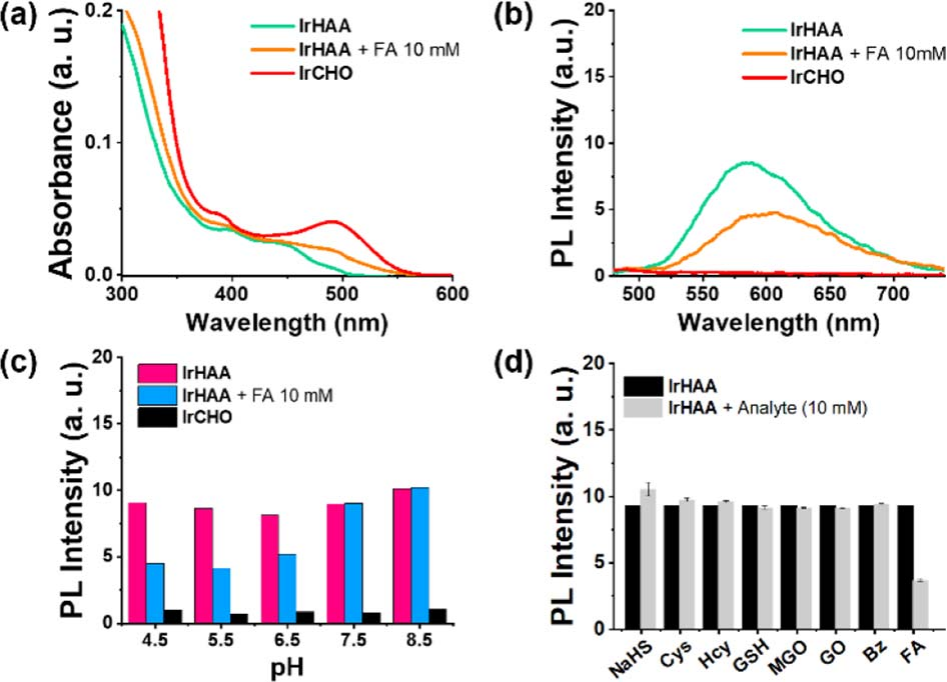
(a) Absorption spectra of iridium complexes (10 μM). (b) Phosphorescence spectra of iridium complexes (10 μM). (c) pH-dependence of phosphorescent detection of formaldehyde (FA) using iridium complexes. (d) Phosphorescence intensity changes of IrHAA with the addition of various analyte (5 mM). Condition: 1 : 1 (v/v) mixture of CH_3_CN and 10 mM HOAc/NaOAc solution (pH 5.0). Excitation at 400 nm.

Fig. 1 shows the photophysical properties of iridium complexes in a 1 : 1 (v/v) mixture of CH_3_CN and 10 mM HOAc/NaOAc aqueous solution. In the UV-vis spectra, IrCHO showed a MLCT band around 500 nm which is 100 nm red-shifted compared to IrHAA, consistent with the density functional theory (DFT) calculation results (Scheme 2). Both IrCHO and IrHAA exhibited broad emission spectra with maximum intensities around 600 nm. Since it is well known that the 2-aza-Cope rearrangement occurs more rapidly under acidic conditions, we checked the reaction of IrHAA with formaldehyde under various pH conditions (Fig. 1c and S1†).^24^ The phosphorescence intensity gradually decreased with the addition of formaldehyde to IrHAA (Fig. S2†). The selectivity test was conducted by comparing the phosphorescence intensities after the addition of various analytes such as biological thiols (Cys = cysteine, Hcy = homocysteine, GSH = glutathione) and reactive carbonyl species (MGO = methyl glyoxal, GO = glyoxal, Bz = benzaldehyde). As expected, other species showed negligible response upon the addition to IrHAA, indicating high selectivity to formaldehyde (Fig. 1d).

**Fig. 2:**
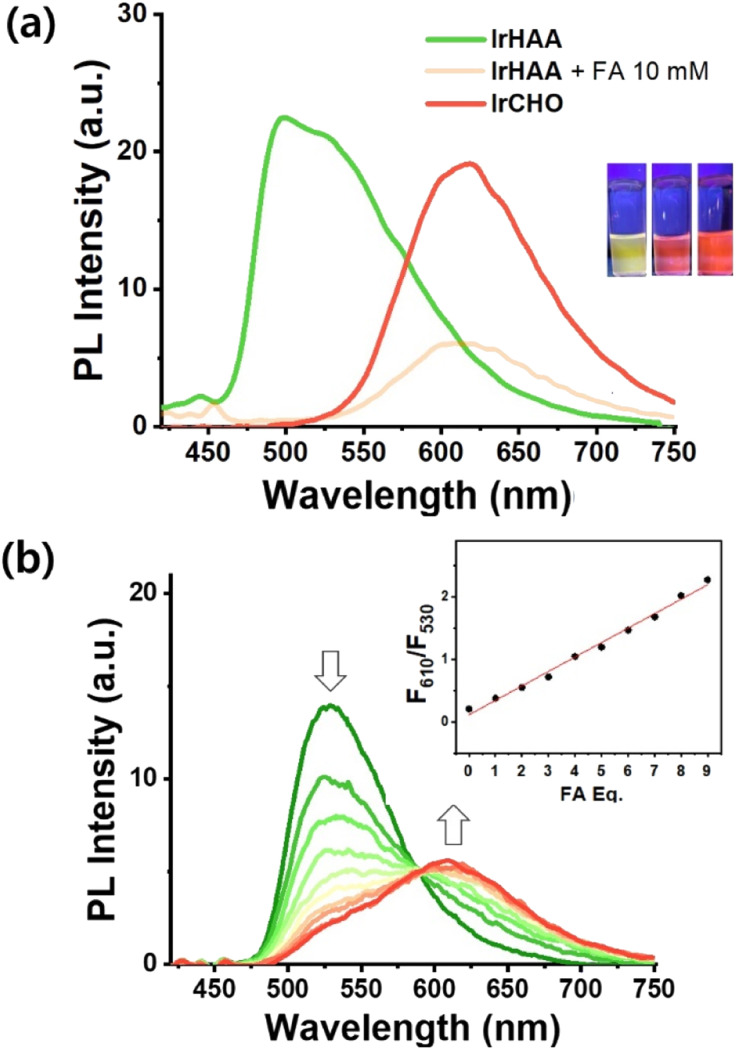
(a) The phosphorescence spectra of 10 μM IrHAA (green) and IrCHO (red). The phosphorescence spectrum of 10 μM IrHAA was recorded 1 hour after the addition of 10 mM formaldehyde (orange). The photographs of samples were taken under irradiation of UV-hand lamp. (b) The phosphorescence intensity changes of IrHAA (10 μM) were recorded 10 hours after each addition of 1 to 10 equivalents of formaldehyde, along with the corresponding linear fitting curve (inset). Condition: CH_3_CN (0.1% TFA).

The presence of a small amount of water had a pronounced quenching effect on the phosphorescence of IrCHO (Fig. S3†). Alternatively, we examined the changes in the photoluminescence intensity using trifluoroacetic acid (TFA) as an acidic medium to take advantage of the relatively high phosphor intensity of iridium complexes in CH_3_CN (Fig. 2). The reaction was completed within 1 hour when a large amount of formaldehyde (10 mM) was added (Fig. S4†). The reaction time was prolonged as the concentration of formaldehyde decreased (Fig. S5†). In CH_3_CN, the addition of formaldehyde to IrHAA showed a clear ratiometric change (Fig. 2a). A good linear calibration curve was obtained by plotting the phosphorescence intensity ratio (*F*_610nm_/*F*_530nm_) against the concentration of formaldehyde within the range of 1 to 10 equiv. (Fig. 2b, inset). The limit of detection (S/N = 3) for formaldehyde was determined to be 0.5 μM.

### Electrochemical properties of iridium complexes

**Fig. 3:**
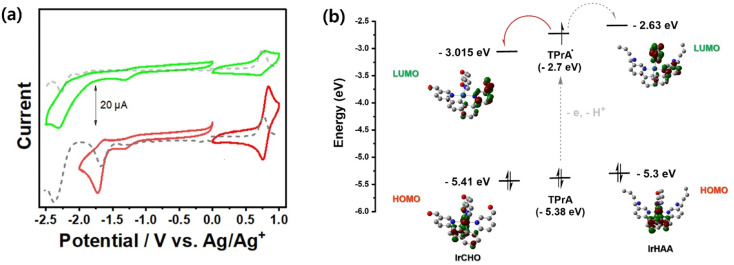
(a) Electrochemical behaviors measured by cyclic voltammetry (solid) and differential pulse voltammetry (dashed) of IrHAA (1 mM, green) and IrCHO (1 mM, red). Condition: CH_3_CN, 0.1 M TBAP as electrolyte, scan rate: 0.1 V s^−1^. (b) Comparison of energy levels of iridium complexes with the SOMO energy level of a TPrA radical. Energy levels of iridium complexes were calculated from those redox potentials which are referenced to Fc/Fc^+^ with equation: *E*_HOMO/LUMO_ (eV) = −e[(*E*_(ox/red)_ − *E*_(Fc/Fc^+^)_)] − 4.80 eV.

We investigated the electrochemical properties of the iridium complexes using cyclic voltammetry (Fig. 3a). Upon scanning the negative potential, irreversible one-electron reduction waves were observed in both IrHAA and IrCHO. The reduction potential of IrCHO (−1.68 eV) showed dramatic anodic shift (390 mV) relative to that of IrHAA (−2.07 eV). The LUMO of IrHAA is mostly located on one pyridyl ring and a picolinate ancillary ligand, while that of IrCHO is mostly localised on only one pyridyl ring (Fig. 3b). The strong electron withdrawing formyl group of IrCHO greatly stabilizes the pyridine ring of the iridium complex and leads to a higher reduction potential compared to IrHAA. The exact potential values were measured using differential pulse voltammetry (Fig. 3a) and converted to the LUMO energy levels using Fc/Fc^+^ as the reference (Fig. 3b). Those energy levels were compared with the SOMO energy level of the TPrA radical, and it was expected that the electron transfer from the TPrA radical to the iridium complex would be favourable after reaction with formaldehyde (Fig. 3b). On the other hand, the oxidation potential values of both IrHAA and IrCHO are similar (Table 1), because the highest occupied molecular orbital of both iridium complexes is mostly distributed in the same phenyl moiety of the main ligand.

**Table 1:** Photophysical and electrochemical properties and quantum efficiencies of iridium complexes and Ru(bpy)_3_Cl_2_ in CH_3_CN

	*λ*_max_ (nm)	*E*_ox_ (V)	*E*_red_ (TPrA˙)	*E*_es_ (eV)	Δ*G* (eV)	*Φ*_PL_ (%)	Rel. ECL int.^b^	Rel. *Φ*_ECL_^c^
Rubpy_3_Cl_2_	611	1.34	−2.1 eV *vs.* (Fc/Fc^+^)	1.99	−1.44	1.60^a^	1.0	1.0
IrHAA	530	0.788		2.34	−0.548	2.81	0.028	0.02
IrCHO	610	0.784		2.03	−0.854	1.53	0.18	0.16

a Obtained from *Chem.–Eur. J*, 1997, **3**(5), 706–712 (in aerated CH_3_CN solution).

b The relative maximum ECL intensities were determined using the Ru(bpy)_3_Cl_2_/TPrA system as a reference in CH_3_CN (0.1 M TBAP).

c The relative ECL efficiencies were determined using the Ru(bpy)_3_Cl_2_/TPrA system as a reference 
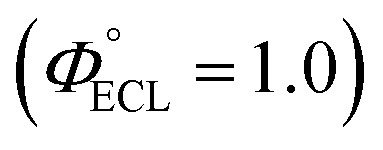
 in CH_3_CN (0.1 M TBAP) according the equation 
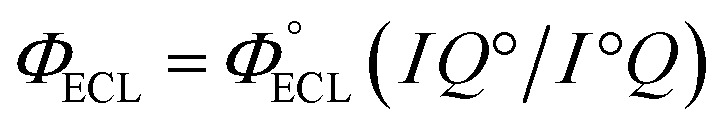
, where *I* and *I*° represent the integrated ECL intensities of the iridium complexes and reference system, respectively, and *Q* and *Q*° are the integrated charges obtained from the corresponding voltammograms of the iridium complexes and reference system, respectively.^8^

### ECL of iridium complexes and formaldehyde detection

**Fig. 4:**
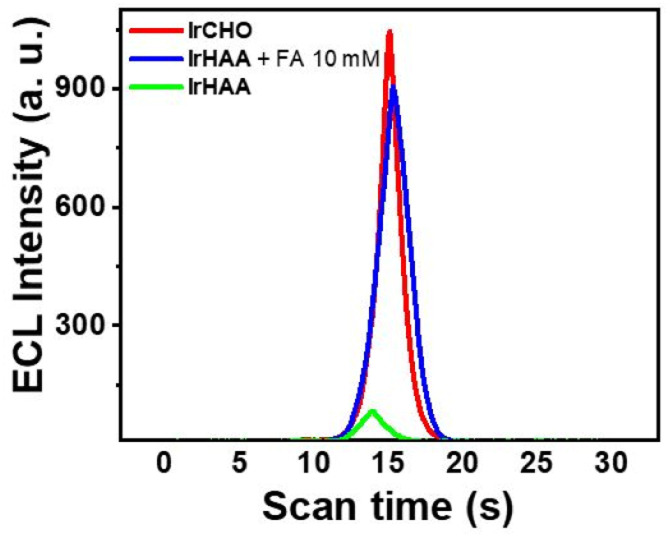
The ECL intensities of iridium complexes (10 μM) as the potential is swept at a working electrode. Condition: CH_3_CN with 0.1% TFA, TPrA (50 mM) as a co-reactant and 0.1 M TBAP as a supporting electrolyte, swept potential: 0–1.5 V, scan rate: 0.1 V s^−1^.

**Fig. 5:**
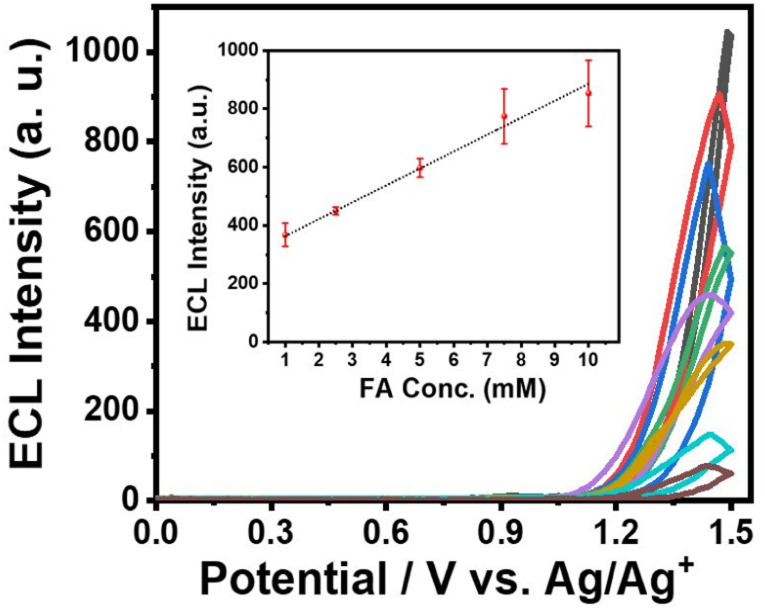
The ECL intensity changes of IrHAA (25 μM) 1 hour after each addition of increasing concentrations of formaldehyde (1, 2.5, 5, 7.5, and 10 mM) and the corresponding linear fitting curve (inset). Condition: CH_3_CN with 0.1% TFA, 0.1 M TBAP, TPrA (50 mM), scan rate: 0.1 V s^−1^.

Then, we performed ECL experiments in CH_3_CN containing 0.1% TFA. In fact, It has been reported that formaldehyde is electrically oxidized to formic acid, which could act as a co-reactant when hydroxyl radical is present.^25^ In the range of applied potential, however, any noticeable signals for a mixture of IrHAA (10 μM) and formaldehyde (10 mM) were not detected in the absence of TPrA in both CV and ECL experiments (Fig. S6†). The ECL of IrHAA itself in the presence of TPrA as the co-reactant was very weak, but dramatically enhanced after the addition of formaldehyde (Fig. 4). When 10 mM of formaldehyde was added to IrHAA, the ECL intensity of the mixture was similar to that of IrCHO. To optimize the conditions for ECL experiments, the relationship between the applied potential and ECL intensity was investigated (Fig. S7†). It was observed that the ECL intensity increased with higher oxidation potentials, indicating that the more oxidized forms of the iridium complex and TPrA were involved in the electrochemical reaction, leading to higher ECL intensity. When the concentration of TPrA was kept constant, the ECL intensity of IrHAA gradually increased with an increasing applied potential. However, the ECL intensity after the addition of 10 mM formaldehyde reached its maximum value at an applied potential of 1.5 V. Therefore, the highest sensitivity (or turn-on ratio) was obtained when the voltage was applied in the range of 0 to 1.5 V. We also investigated the optimal concentration of TPrA (Fig. S8†). If the concentration of TPrA is too low (10 mM), it may be insufficient to effectively participate in the electrochemical reaction, resulting in poor ECL efficiency. On the other hand, an excessively high concentration of TPrA (100 mM) can lead to lower ECL intensity. These effects can decrease the ECL intensity and reduce the reproducibility of ECL in repeated measurements. Comparisons among the different concentrations of TPrA revealed that all iridium complexes exhibited higher ECL intensities at 25 mM of TPrA. However, the relative turn-on ratio at this concentration was low, showing only a 2-fold increase. Conversely, even though the addition of 50 mM of TPrA resulted in lower ECL intensities, the turn-on ratio significantly increased by 8-fold. We measured the ECL intensities using 50 mM of TPrA as a co-reactant upon the addition of an increasing amount of formaldehyde to IrHAA (Fig. 5). The maximum ECL intensity at 1.5 V gradually increased with the addition of formaldehyde in the range of 1 to 10 mM. The ECL intensity upon the addition of 10 mM formaldehyde was 8-fold higher compared to that of IrHAA. We obtained a linear relationship between the maximum ECL intensities and the concentration of added formaldehyde.

**Fig. 6:**
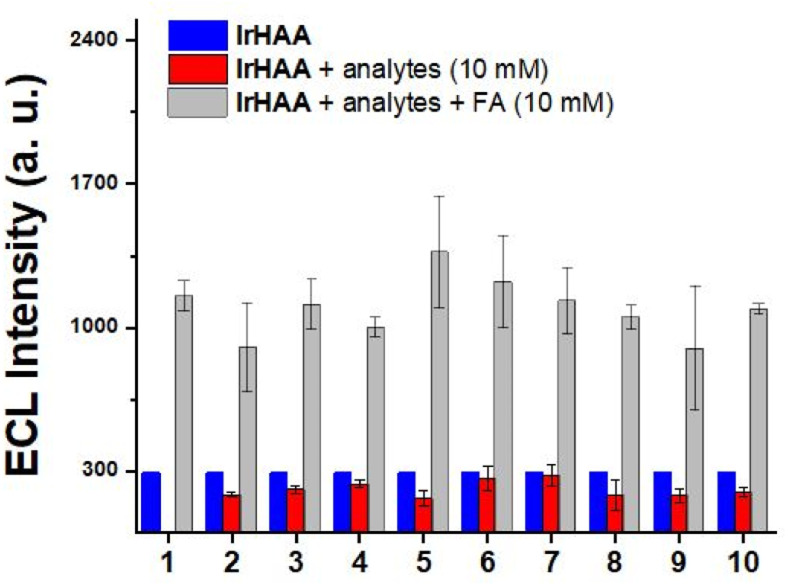
ECL response of IrHAA (25 μM) before and after addition of various analytes (10 mM), (1) formaldehyde (FA) only, (2) glyoxal, (3) methyl glyoxal, (4) acetaldehyde, (5) benzaldehyde, (6) benzoic acid, (7) formic acid, (8) acetylacetone, (9) acrolein, (10) vanillin. Condition: CH_3_CN with 0.1% TFA, 0.1 M TBAP, TPrA (50 mM), scan rate: 0.1 V s^−1^.

We also performed a competitive assay using ECL with a range of carbonyl species, including various aldehyde, ketone, and carboxylic acid derivatives (Fig. 6). These compounds having moderate solubility in CH_3_CN showed negligible responses in ECL upon reacting with IrHAA. However, further addition of formaldehyde enhanced the ECL intensities of all the analytes to the extent comparable to when only formaldehyde was added. Consequently, IrHAA showed high selectivity towards formaldehyde in ECL detection.

### Energetic requirement (driving force)

The ECL efficiency of iridium complexes is determined by (1) the efficiency of generating excited states by electron transfer from the TPrA radical and (2) the radiative relaxation efficiency (*Φ*_PL_) of iridium complexes.^14,26,27^ Hogan and co-workers introduced a simple method to determine the energy requirement (Δ*G*) for the generation of excited states in iridium complexes. This is accomplished by solving the following equation, which relates the oxidation potential 
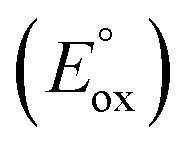
 and excited state energy (*E*_es_) of the complexes:^14^1
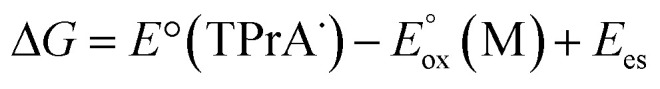


Considering that the reported energy level of the SOMO of the TPrA radical, *i.e.*, *E*°(TPrA˙), is −2.1 V *vs.* Fc/Fc^+^ in CH_3_CN,^27^ substituting the measured oxidation potential and excited state energy into eqn (1) allows for the calculation of the Gibbs free energy values (Δ*G*) for each iridium complex (Table 1). As expected, the Δ*G* value of IrCHO is more negative compared to that of IrHAA. This indicates that IrCHO forms excited states more efficiently through electron transfer. We also obtained the luminescent quantum yields (*Φ*_PL_) and ECL efficiencies (*Φ*_ECL_) of the iridium complexes. As a result, the ECL efficiency of IrCHO was found to be 8 times higher than that of IrHAA, albeit its lower *Φ*_PL_.

### Mechanism study

In fact, some iridium complexes are unstable and decomposed under acidic conditions. Therefore, it is crucial to identify the actual product formed after the reaction with formaldehyde, especially in cases where such conditions are involved.

We performed combined analysis using NMR and MALDI-TOF experiments to gain insights into the reaction between formaldehyde and IrHAA. Our NMR results (Fig. S9†) clearly indicate the presence of formyl proton peaks at approximately 10.0 ppm in IrCHO, which are distinct from the proton peak of formaldehyde itself. When formaldehyde is added to IrHAA in the presence of 0.1% TFA, the formyl proton peaks observed are identical to those in IrCHO. Furthermore, MALDI-TOF analysis (Fig. S10†) revealed the formation of imine intermediates upon the addition of formaldehyde to IrHAA. However, it is important to note that the subsequent 2-aza-Cope rearrangement reaction did not occur in the absence of an acidic medium (Fig. S9†). This underscores the critical role of an acid catalyst in facilitating the 2-aza-Cope rearrangement reaction.

## Conclusions

In this study, we report a molecular probe based on an iridium complex (IrHAA) that enables the detection of formaldehyde through both phosphorescence and ECL. The detection mechanism relies on the modulation of the electron density of the LUMO through the 2-aza-Cope rearrangement of the reaction adduct formed between IrHAA and formaldehyde. Modulating the electronic structure and energy levels of an iridium complex through a reaction-based approach offers a powerful strategy for designing turn-on ECL molecular probes. We are currently developing an iridium complex-based probe showing excellent phosphorescence and ECL performance in aqueous solution for monitoring formaldehyde in real samples, including river water, human serum, and urine.

## Conflicts of interest

There are no conflicts to declare.

## Supplementary Material

RA-013-D3RA06936B-s001
