# Supplementary material for: Reaction-based energy level modulation of a cyclometalated iridium complex for electrochemiluminescent detection of formaldehyde
Source: RSC Adv. 2023 Nov 1;13(46):32070–6. doi: 10.1039/d3ra06936b (PMC10618939; doi:10.1039/d3ra06936b)
Supplement: RA-013-D3RA06936B-s001 [file RA-013-D3RA06936B-s001.pdf]

Electronic Supplementary Material (ESI) for RSC Advances.
This journal is © The Royal Society of Chemistry 2023

Electronic Supplementary Material (ESI) for

Reaction-based energy level modulation of a cyclometalated iridium complex for electrochemiluminescent detection of formaldehyde

Hyun Seung No and Jong-In Hong*

Department of Chemistry, College of Natural Sciences, Seoul National University, Seoul 08826, Republic of Korea

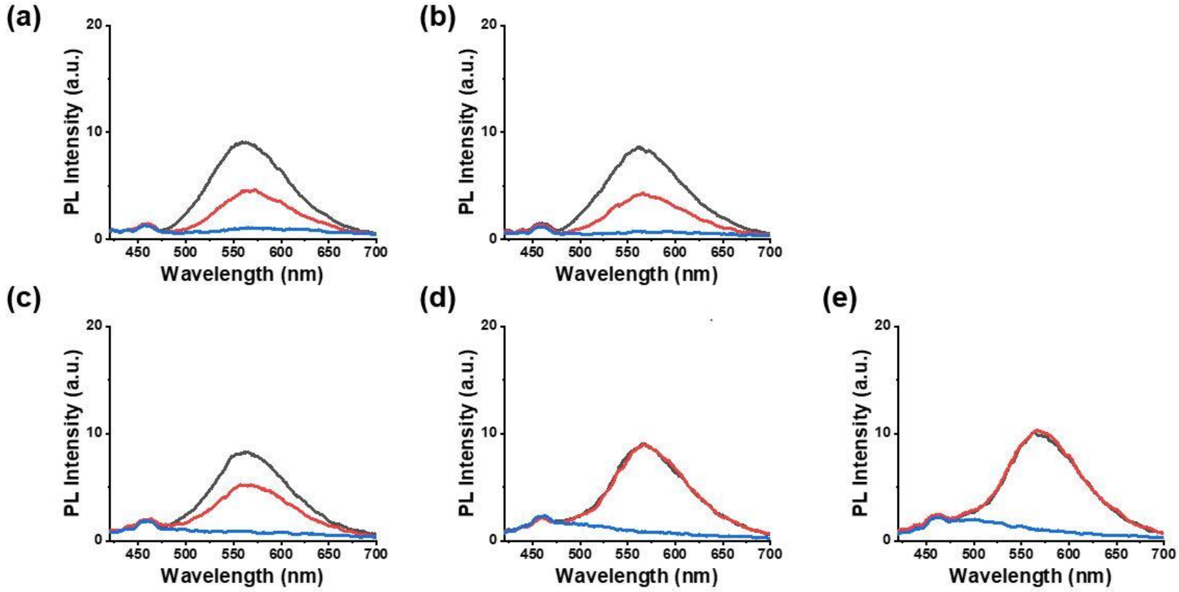

Figure S1. pH-dependent phosphorescence spectra of **IrHAA** (Black), **IrHAA** + formaldehyde (FA) 10 mM (Red), **IrCHO** (Blue). Adjusted pH: a) 4.5, b) 5.5, c) 6.5, d) 7.5, e) 8.5.

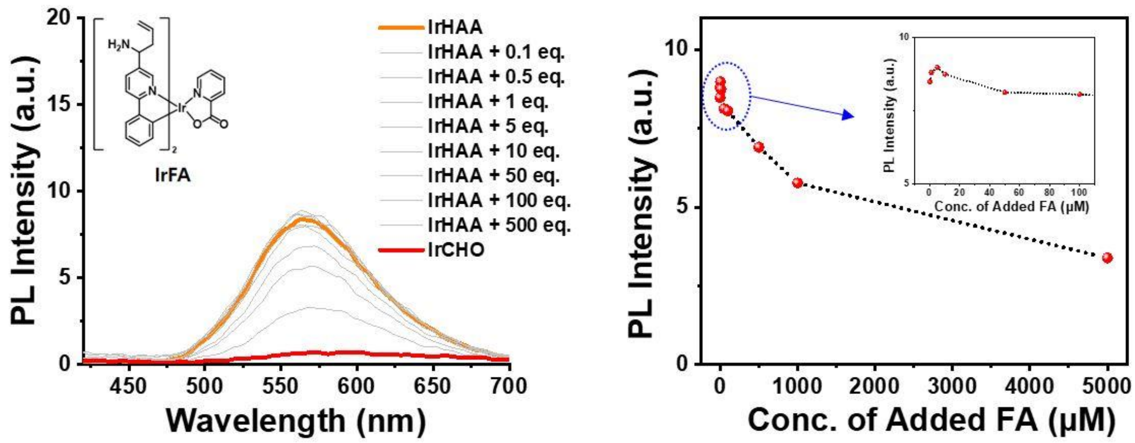

Figure S2. (Left) Phosphorescence spectra of **IrHAA** (10 μM) with the addition of FA in aqueous solution and (Right) corresponding calibration curve.

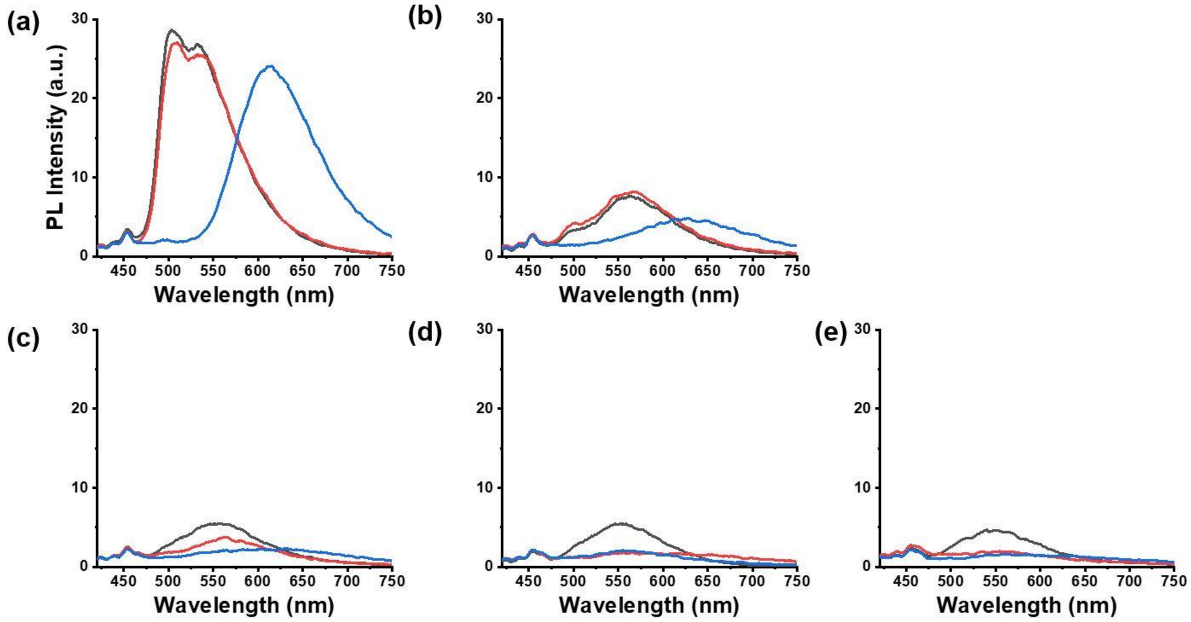

Figure S3. Phosphorescence spectra of **IrHAA** (Black), **IrHAA** + FA 10 mM (Red), and **IrCHO** (Blue) in CH_3CN/H_2O (v/v) mixture with different ratios (v/v = (a) 10:0, (b) 9:1, (c) 8:2, (d) 7:3, (e) 6:4).

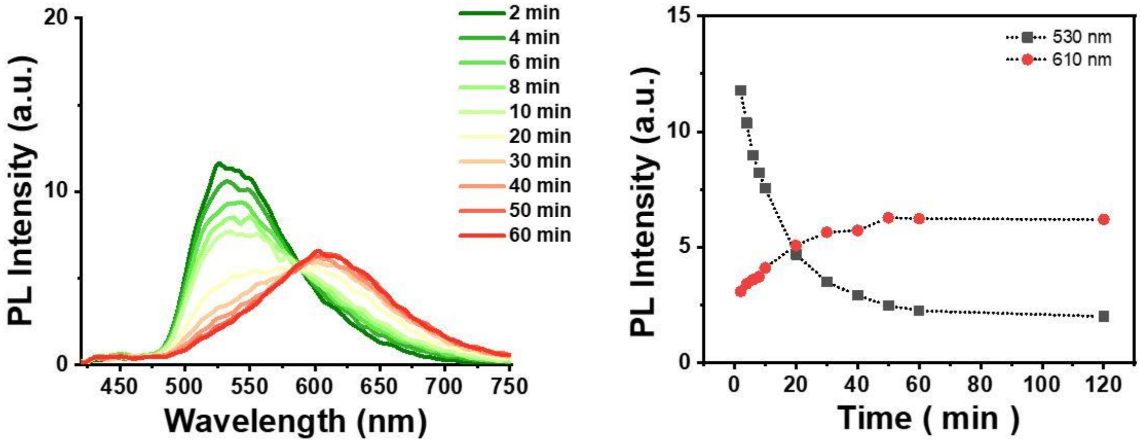

Figure S4. (a) Time-dependent phosphorescence spectral changes of **IrHAA** with the addition of FA (10 mM) in CH_3CN with 0.1% TFA.

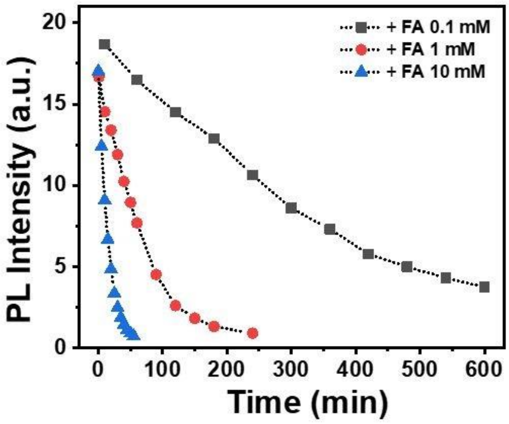

Figure S5. Changes in the time-dependent phosphorescence intensity of **IrHAA** with the addition of various concentrations of FA (0.1, 1, 10 mM) in CH_3CN solution with 0.1% TFA.

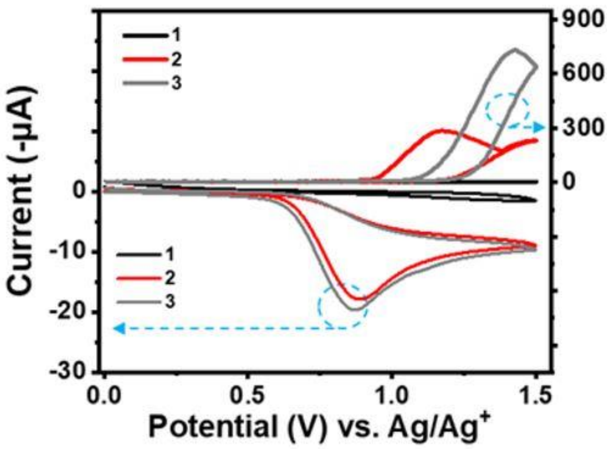

Figure S6. Cyclic voltammograms (bottom) and corresponding ECL intensities (top) measured during potential sweep (0 ~ 1.5 V) in CH_3CN (0.1% TFA) in the presence of 0.1 M TBAP after the addition of (1) **IrHAA** (10 μM) + FA (10 mM), (2) **IrHAA** (10 μM) + TPrA (20 mM), (3) **IrHAA** (10 μM) + FA (10 mM) + TPrA (20 mM).

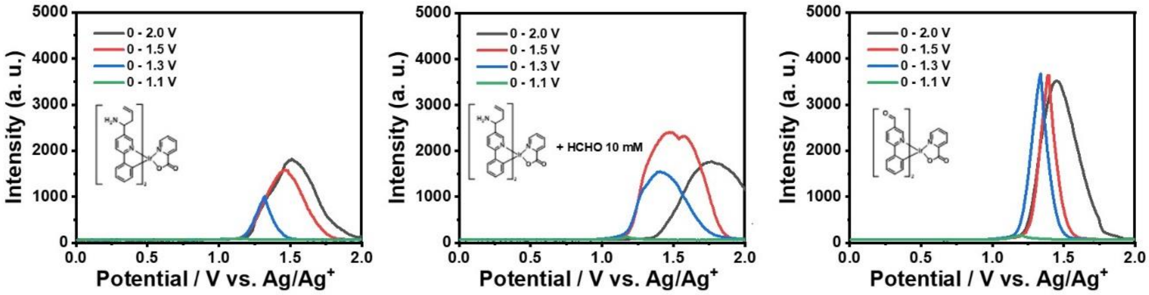

Figure S7. ECL intensities of iridium complexes (10 μM) under different sweep oxidation potential (0 ~ 1.1, 1.3, 1.5 and 2.0 V) with TPrA as the co-reactant (20 mM).

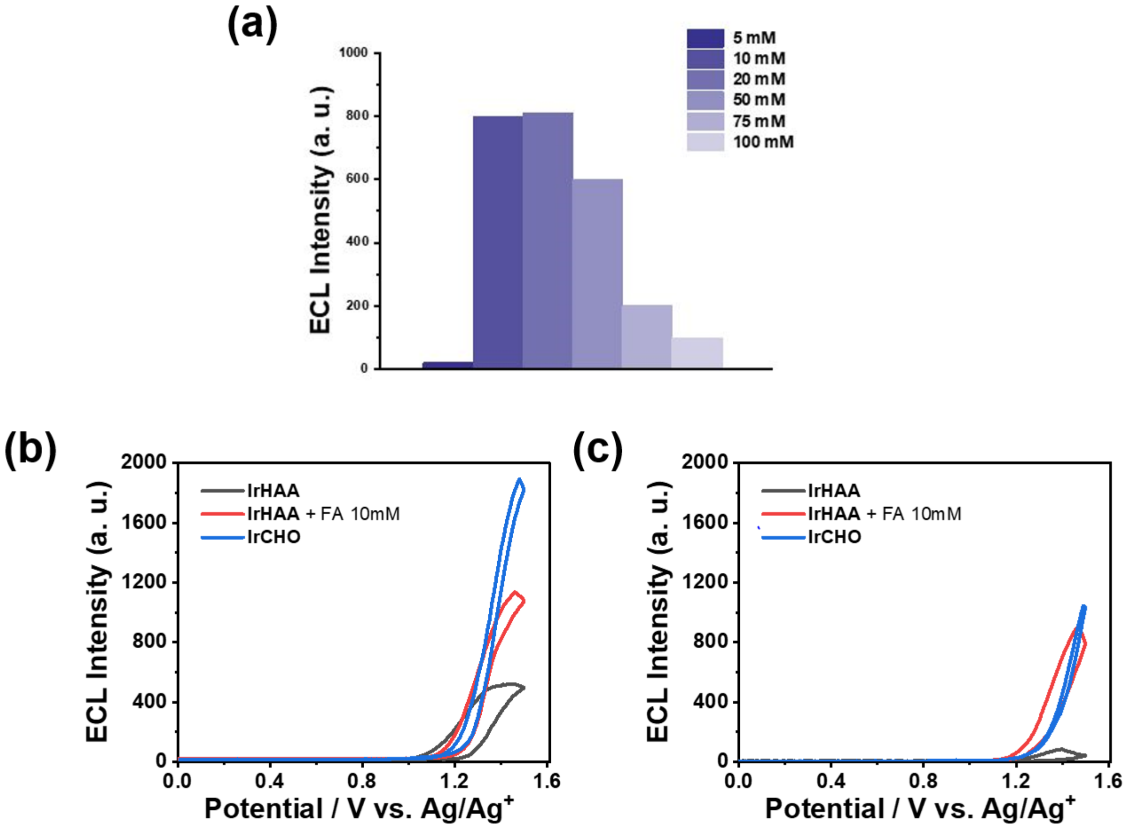

Figure S8. (a) Comparison of ECL intensities of **IrHAA** (25 μM) + FA (10 mM) under different concentrations (5, 10, 20, 50, 75, 100 mM) of TPrA, (b) Comparison of ECL intensities of iridium complexes (25 μM) after addition of 20 mM of TPrA, (c) Comparison of ECL intensities of iridium complexes after addition of 50 mM of TPrA.

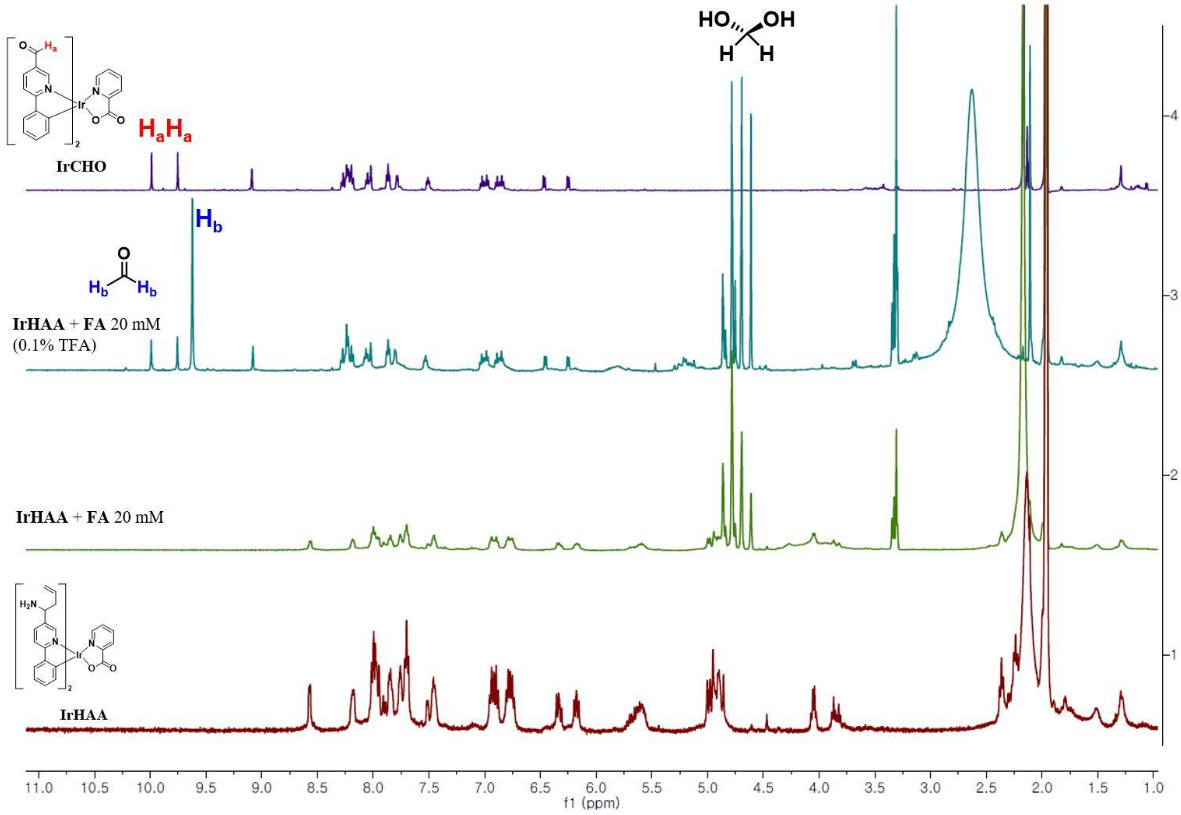

Figure S9. Comparison of ^{1}H NMR spectra of **IrHAA** (2 mM) before and after the addition of FA (20 mM) with **IrCHO** (2 mM) in CD_3CN.

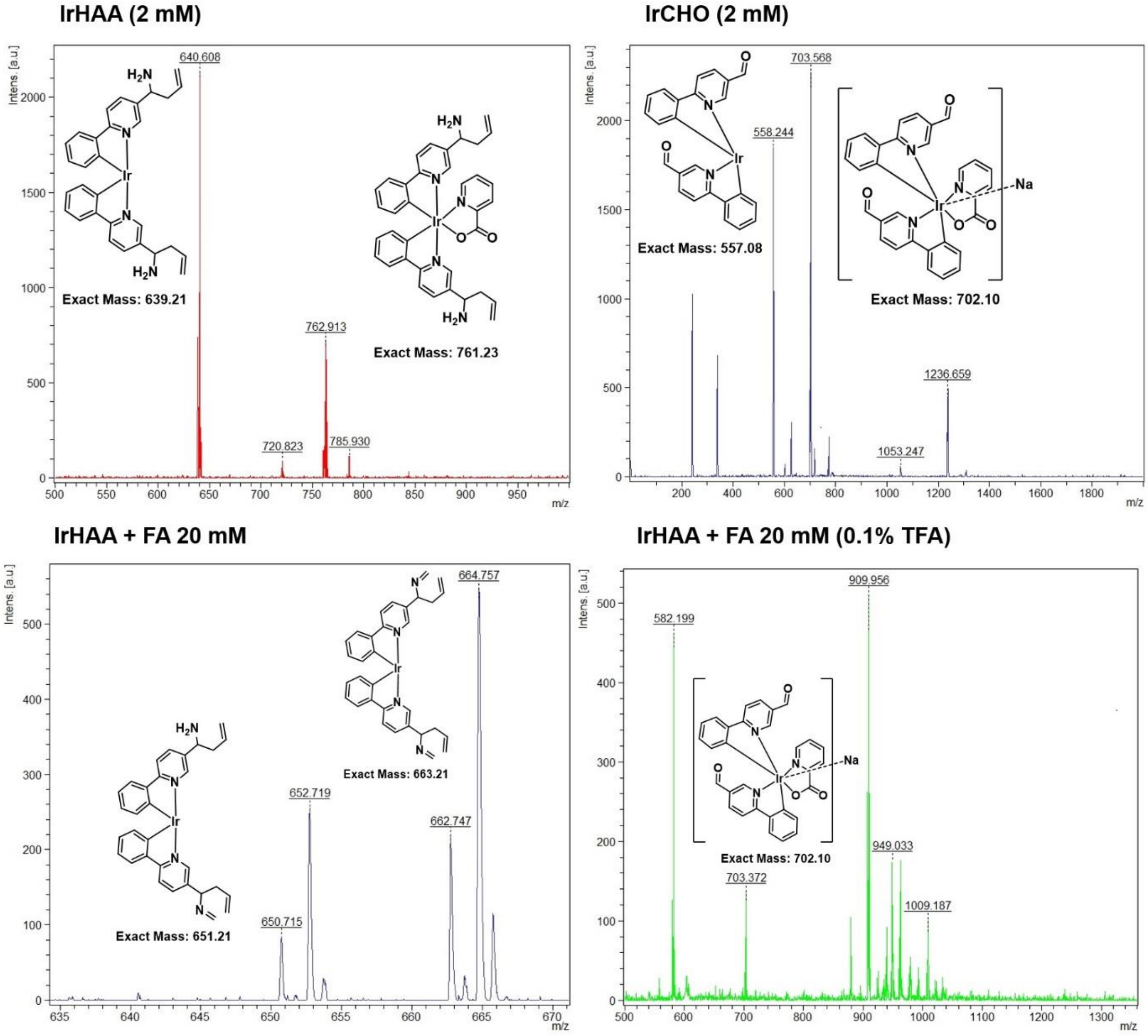

Figure S10. MALDI-TOF mass spectra of (a) **IrHAA** (2 mM), (b) **IrCHO** (2 mM), (c) **IrHAA** with the addition of FA, (d) **IrHAA** with the addition of FA and 0.1 % TFA in CH_3CN.

^{1}H and ^{13}C NMR

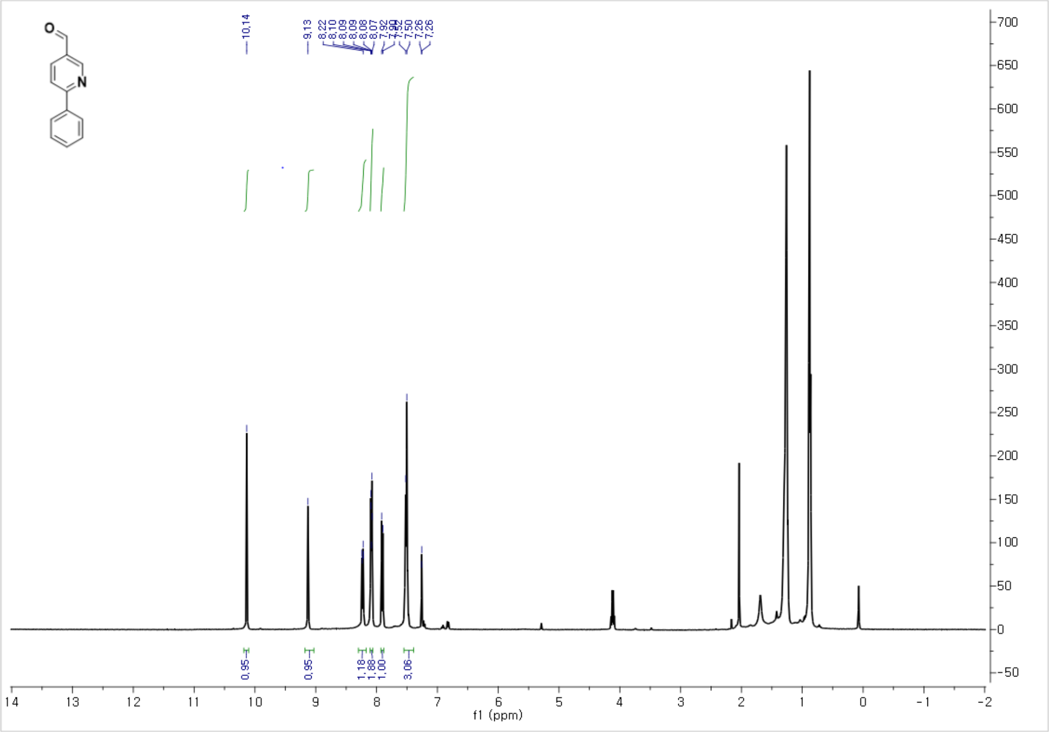

Figure S11. ^{1}H NMR of 6−phenyl−3−pyridinecarboxaldehyde (400 MHz, $CDCl_3$).

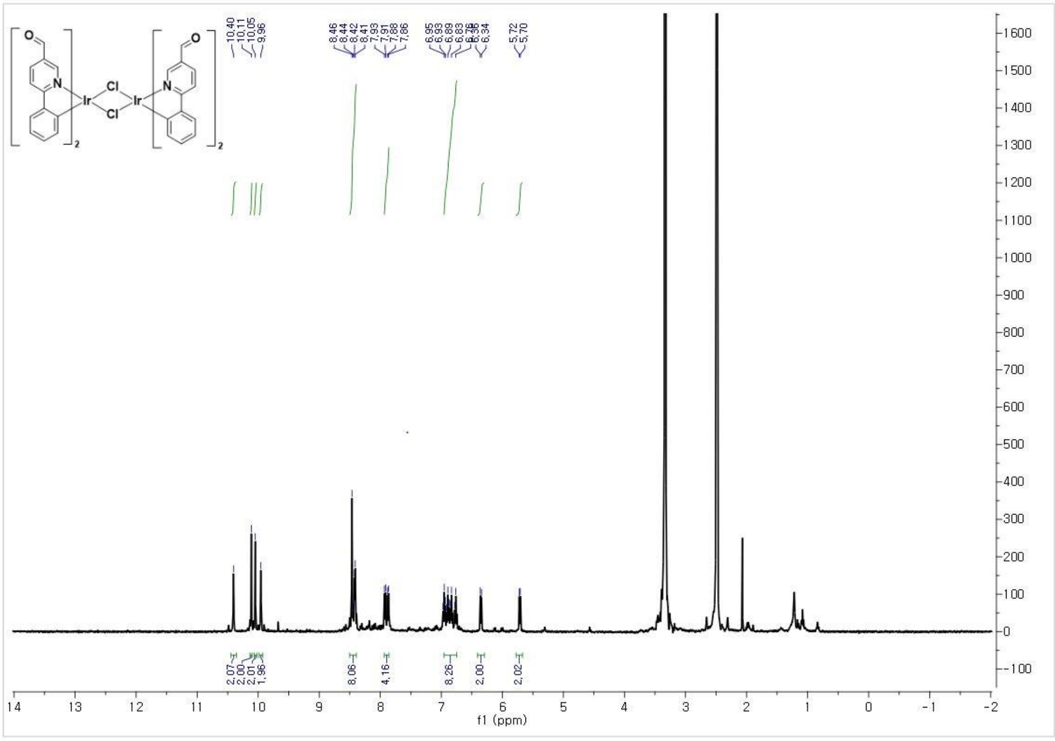

Figure S12. ^{1}H NMR of iridium dimer complex (400 MHz, DMSO-d_6).

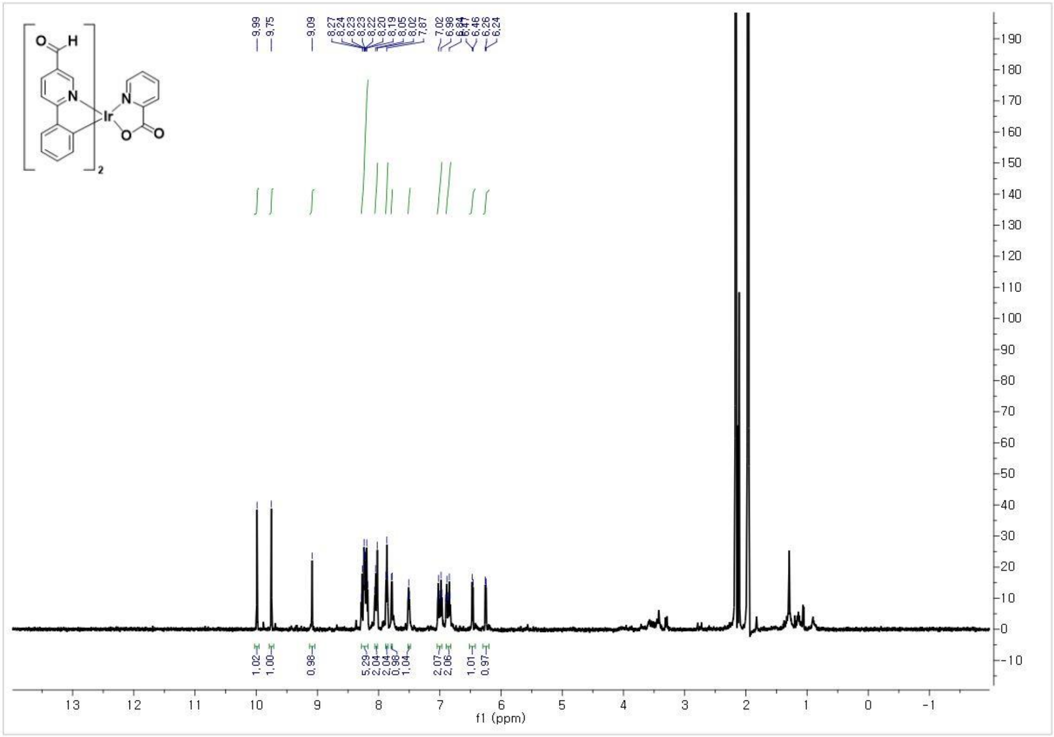

Figure S13. ^{1}H NMR of **IrCHO** (500 MHz, CD_3CN).

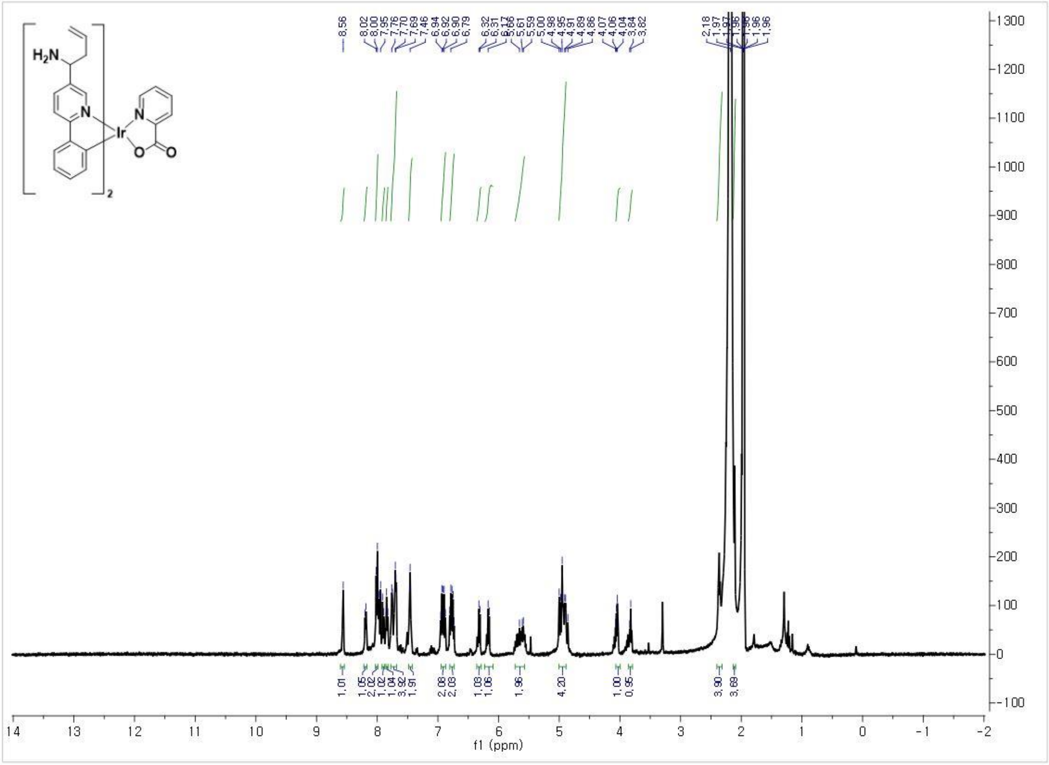

Figure S14. ^{1}H NMR of **IrHAA** (400 MHz, CD_3CN).

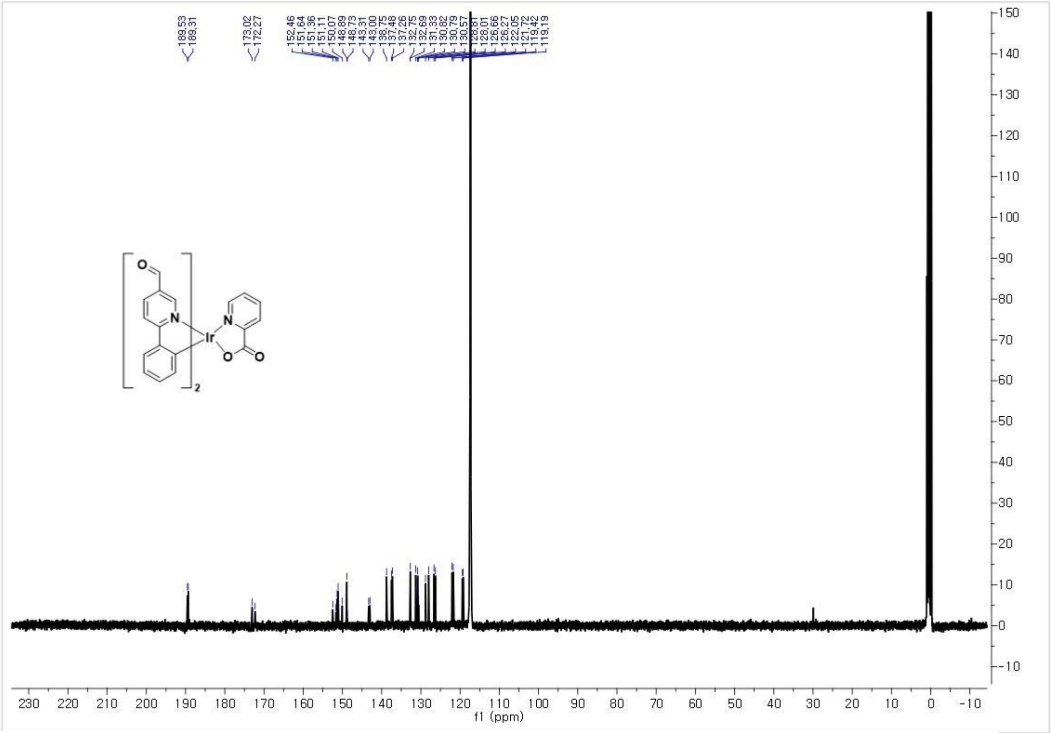

Figure S15. ^{13}C NMR of **IrCHO** (101 MHz, CD_3CN).

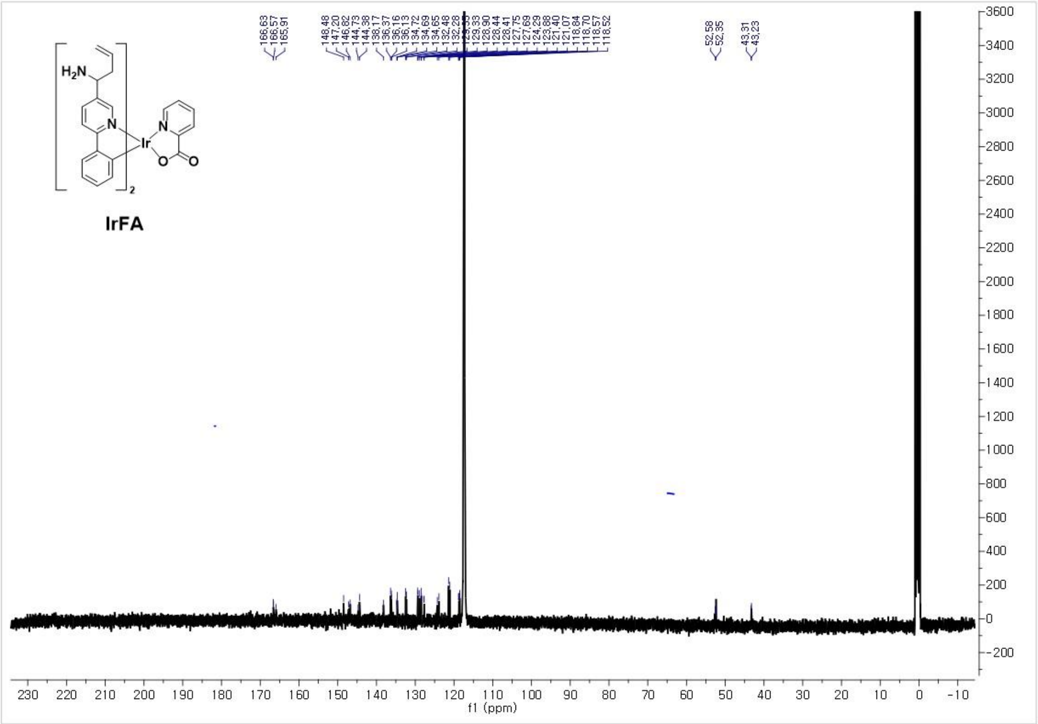

Figure S16. ^{13}C NMR of **IrHAA** (101 MHz, CD_3CN).

High Resolution Mass Spectra (HRMS)

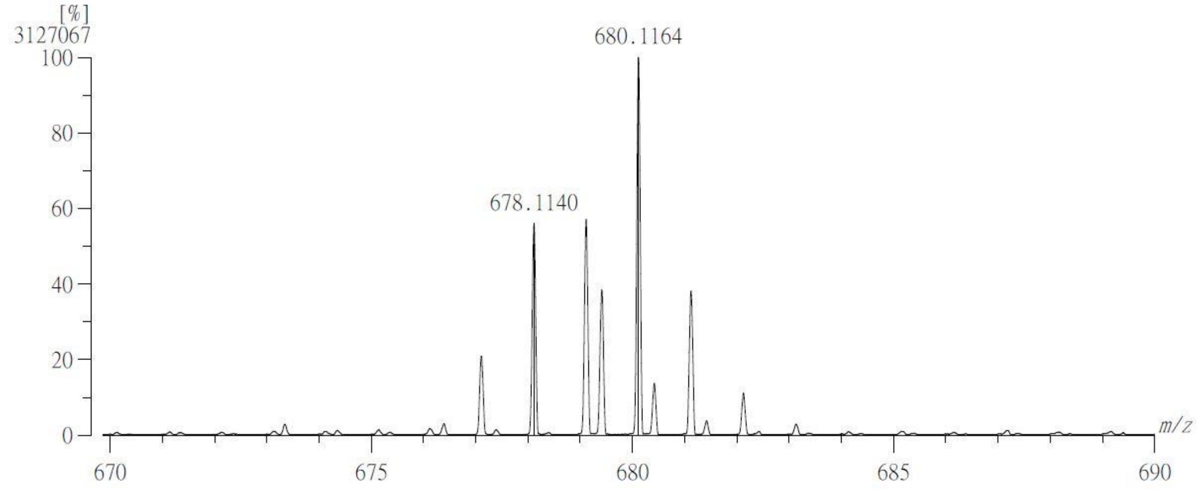

Figure S17. HRMS of **IrCHO**.

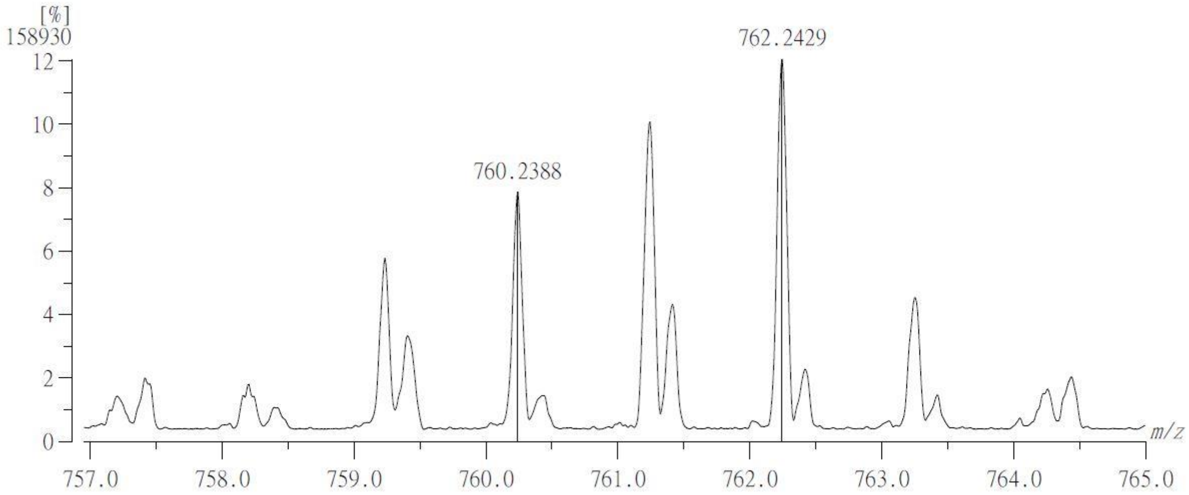

Figure S18. HRMS of **IrHAA**.
